# Preliminary Assessments of Phenolic Profiles, Antioxidant Capacities, and Antiviral Activities of Fifteen Rosaceae Species

**DOI:** 10.3390/molecules31203651

**Published:** 2026-10-13

**Authors:** Serkos A. Haroutounian, Anna Apostolou, Lieve Naesens, Epameinondas Evergetis, Sandra Liekens, Eleni D. Myrtsi

**Affiliations:** 1 Laboratory of Nutritional Physiology & Feeding, Department of Animal Science, Agricultural University of Athens, Iera Odos 75, 11855 Athens, Greece; sehar@aua.gr (S.A.H.); annapost21@msn.com (A.A.); 2 Rega Institute for Medical Research, KU Leuven, Herestraat 49, 3001 Leuven, Belgium; lieve.naesens@kuleuven.be (L.N.); sandra.liekens@kuleuven.be (S.L.)

**Keywords:** Rosaceae, Amygdaloideae, Rosoideae, flavonoids, phenolics, antioxidant, antiviral

## Abstract

The increasing prevalence of antiviral drug resistance is limiting the number of available therapies, prompting a need to introduce novel antiviral compounds. Because nature represents a rich reservoir of bioactive molecules, a broad spectrum of research endeavors has been initiated for the exploitation of plant-origin extracts. In this context, the methanolic extracts of the aerial parts of 15 Rosaceae plant species belonging to the Amygdaloideae and Rosoideae subfamilies were investigated with regard to their phenolic content, antioxidant capacity and antiviral properties. Their total phenolic and flavonoid contents were determined, and the presence of individual phenolic compounds was quantified by LC–MS/MS analyses. Additionally, the antioxidant capacities of extracts were evaluated utilizing ABTS and DPPH assays, while antiviral properties were assessed against a broad panel of DNA and RNA viruses. All investigated extracts displayed strong antioxidant potency, as well as substantial variation in their phenolic profiles, with *Prunus webbii* exhibiting the highest total phenolic content (541.3 mg GAE/g DWE) and *Sorbus umbellata* exhibiting the highest total flavonoid content (116.1 mg QE/g DWE). With regard to the presence of individual phenolics, chlorogenic acid was the prevailing molecule in Amygdaloideae subfamily plants, with *Pyrus spinosa* displaying the highest content (35,173.6 mg/100 g DWE), while ellagic acid was the most abundant phenolic in Rosaceae plants, with *Potentilla speciosa* exhibiting the highest concentration (4127.0 mg/100 g DWE). All extracts exhibited notable antiviral activity against one or more of the tested virus strains. The lowest IC_50_ values were recorded against the influenza A and B strains, with the *Sorbus Umbellata* extract displaying an IC_50_ value of 0.8 μg/mL across all tested influenza strains. The *Potentilla recta*, *Crataegus orientalis* and *Pyracantha coccinea* extracts exhibited specific activity against the influenza A (H3N2) strain, with IC_50_ values of 0.4, 0.6 and 0.8 μg/mL, while *Pyracantha coccinea* extract was active against the influenza A (H1N1) strain, with an IC_50_ value of 1.6 μg/mL. These preliminary findings provide initial evidence of antiviral and antioxidant activity of the investigated Rosaceae extracts that warrant further investigation.

## 1. Introduction

Viral infectious diseases pose a major global health challenge which is continuously being exacerbated by the emergence of new pathogens, the rapid evolution of viruses, and the increasing prevalence of antiviral drug resistance. This problem has been further intensified by a simultaneous increase in phenomena such as drug resistance, an increase in the spread of new diseases, and the limited availability of effective antiviral agents. Consequently, several viruses, including herpesviruses, adenoviruses, and influenza viruses, have emerged as major causes of various human diseases. Additionally, many viral pathogens evolve rapidly through mutation, reducing the long-term effectiveness of antiviral therapies and increasing the likelihood of recurrent epidemics or the emergence of novel outbreaks [1]. These challenges underscore the urgent need for the discovery and development of novel, potent antiviral agents. Natural products inherently play a significant role in the design and development of novel therapeutics, because their enormous structural diversity can provide high screening hit rates against various diseases and viruses [2]. Thus, the identification of new bioactive compounds or extracts from natural products can provide structurally diverse and mechanistically novel candidates capable of overcoming the resistance problem, improving selectivity, enhancing therapeutic effectiveness, and strengthening global health preparedness through advancement of medical innovation. For this purpose, several plant secondary metabolites have been utilized as valuable agents for the treatment and prevention of various infections and diseases. Among these molecules, the phenolic compounds have attracted increasing interest as antiviral agents because of their capability to interfere with multiple stages of viral life cycle, such as viral attachment, entry, replication, and assembly. The structural diversity and multitarget mechanisms of phenolics highlight their use as promising candidates for the development of novel antiviral therapeutics, particularly against various rapidly evolving viruses.

Among the plethora of investigated plant species, various plants belonging to the Rosaceae family have been identified as rich sources of diverse phenolic compounds displaying potent antioxidant and antibacterial activities [3,4]. In particular, plants of the Amygdaloideae subfamily have attracted considerable scientific attention because of their content of amygdalin, a cyanogenic glycoside that has been reported to exhibit notable anticancer and neuroprotective properties [5,6]. Rosoideae constitutes another Rosaceae subfamily that includes several economically important plants such as strawberries (*Fragaria ananassa*), raspberries (*Rubus idaeus*), blackberries (*Rubus fruticosus*) and roses (*Rosa*) [7,8]. Due to their high antioxidant capacities, these plant species are widely used as superfoods and have attracted considerable economic and research interest [7,9]. Consequently, a notable knowledge gap has been identified in the scientific literature between the economically important and the less-highly valued Rosoideae plant species, as most efforts have been disproportionately concentrated on a limited number of species. Thus, numerous plant species belonging to other genera within the same subfamily, such as *Potentilla* spp., remain largely unexplored [10]. To further verify this observation, a recent search of the Scopus database (2 April 2026) indicated the existence of numerous publications concerning various economically important Rosoideae species such as *Fragaria* × *ananassa* and *Rosa* spp., whereas species such as *Potentilla haynaldiana* and *Alchemilla bulgarica* were found to remain almost completely unexplored.

The present study aims to provide a comprehensive evaluation of the phenolic contents and antioxidant activities of methanolic extracts obtained from the aerial parts of fifteen Rosaceae taxa, seven species of which are classified into the Amygdaloideae subfamily, namely, *Pyracantha coccinea* (PyCo), *Pyrus spinosa* (PySp), *Crataegus orientalis* (CrOr), *Prunus webbii* (PrWe), *Prunus mahaleb* (PrMa), *Sorbus umbellata* (SoUm), *Prunus prostrata* (PrPr), and eight species of which belong to the Rosoideae subfamily, namely, *Potentilla recta* (PoRe), *Potentilla pedata* (PoPe), *Potentilla speciosa* (PoSp), *Potentilla haynaldiana* (PoHi), *Alchemilla bulgarica* (AlBu), *Alchemilla xanthochlora* (AlXa), *Aremonia agrimonoides* (ArAg), and *Geum urbanum* (GeUr).

The main research objectives of this study concern the exploitation of methanolic extracts from selected Rosaceae taxa in order to (i) assess their total phenolic and flavonoid contents; (ii) quantify the presence of selected individual bioactive phenolic molecules; (iii) evaluate their antioxidant capacity with the ABTS and DPPH assays; and (iv) perform an exploratory screening of their antiviral activity against a broad panel of DNA and RNA viruses.

## 2. Results

Because the present work represents an exploratory screening study, all measurements herein were obtained using a single biological replicate per species. Therefore, the results should be interpreted only as a preliminary comparative trend and not as statistically conclusive evidence.

### 2.1. Assessment of Total Phenolic (TPC) and Flavonoid (TFC) Contents

**Figure 1:**
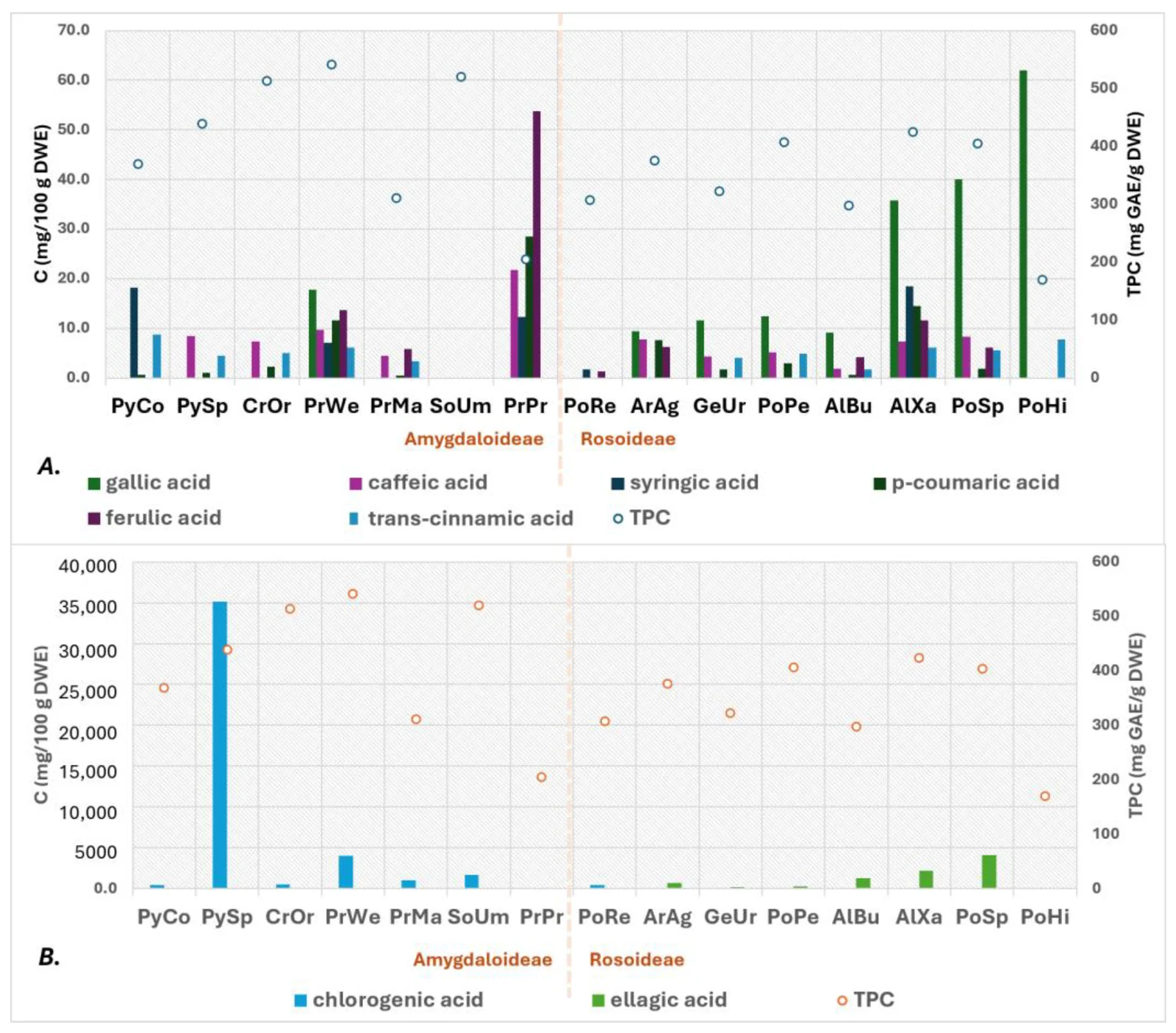
TPC measurement and quantitation of phenolic acids presence (**A**). gallic acid, caffeic acid, syringic acid, *p*-coumaric acid, ferulic acid, and *trans*-cinnamic acid; (**B**). chlorogenic acid and ellagic acid) in the plant species investigated. Concentrations of phenolic acids are expressed as mg/100 g of dried extract and plotted on the primary y-axis, whereas TPC is expressed as mg GAE/g of dried extract and plotted on the secondary y-axis.

**Figure 2:**
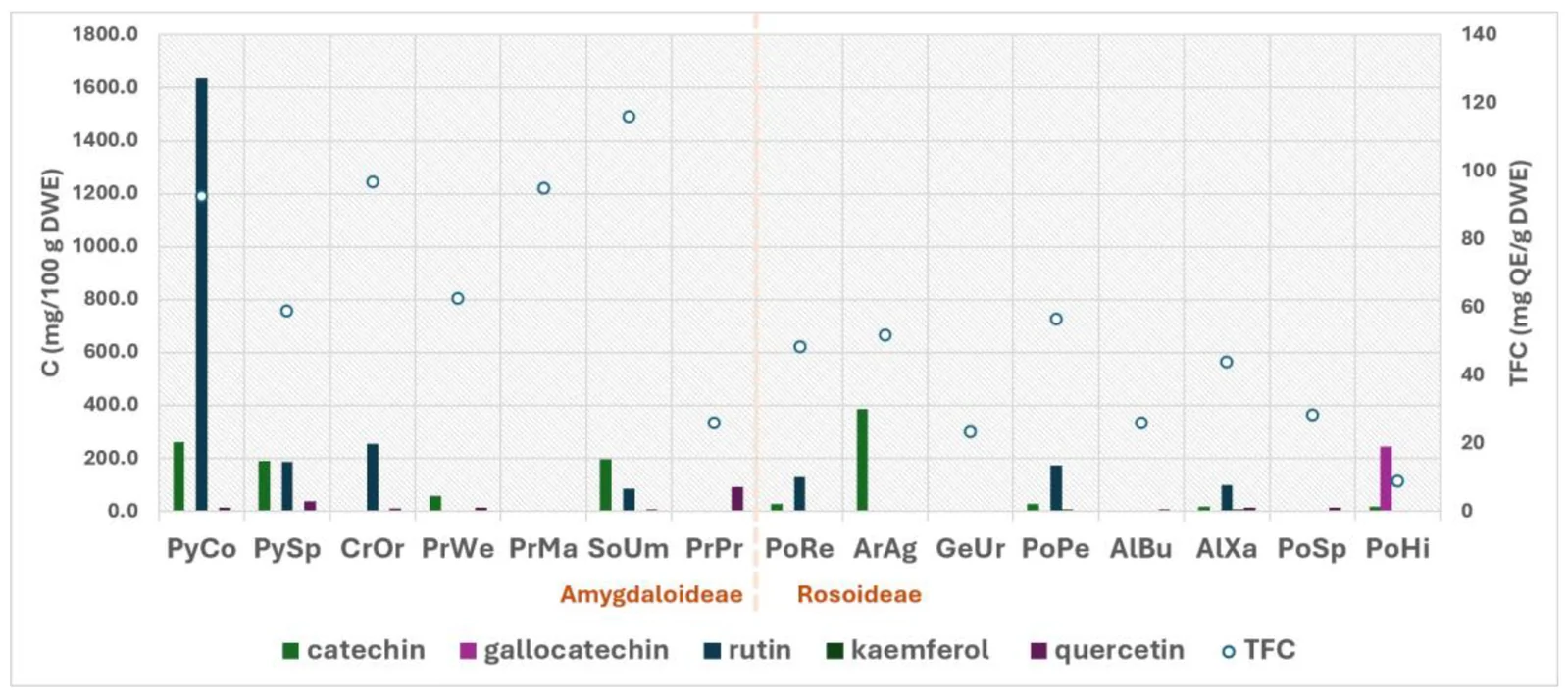
Quantitation of presence of phenolic compounds and TFC in the investigated plant species. Concentrations are expressed as mg/100 g of their dried extract (primary y-axis), and TFC is expressed as mg QE/dried extract (secondary y-axis).

The total phenolic and flavonoid content values assessed herein are included in Table S1 of the Supplementary Materials section and presented in Figure 1 and Figure 2. According to these measurements, the Amygdaloideae subfamily plant extracts exhibited higher TPC and TFC values as compared with the investigated Rosoideae subfamily plant extracts. Specifically, the highest TPC value (541.3 mg GAE/g DWE) was determined for the methanolic extract of PrWe, followed by the extracts of SoUm (520.3 mg GAE/g DWE) and CrOr (513.3 mg GAE/g DWE). Regarding flavonoid content, the SoUm extract exhibited the highest TFC value (116.1 mg QE/g DWE), followed by the CrOr (96.9 mg QE/g DWE) and PrMa (95.0 mg QE/g DWE) extracts. Among the investigated Rosoideae subfamily plants, the methanolic extract of AlXa displayed the richest phenolic content (424.3 mg GAE/g DWE), whereas the PoPe extract showed the highest flavonoid content (56.6 mg QE/g DWE).

### 2.2. Quantitation of Individual Phenolic Compounds Presence by HPLC and LC–MS/MS Analysis

The outcome of quantitating eleven abundant bioactive phenolic compounds is included in Table S2 of the Supplementary Materials section and presented in Figure 1 and Figure 2. The quantitation of phenolic acids (Figure 1) indicated that extracts of Amygdaloideae subfamily plants were characterized by a high concentration of chlorogenic acid, with the PySp extract exhibiting the highest content. On the contrary, the extracts of Rosoideae subfamily plants were determined to contain large amounts of ellagic acid, with PoSp extract displaying the highest content. The methanolic extract of the Amygdaloideae subfamily plant *Prunus prostrata* was the only extract not containing any of these two phenolic acids. Finally, the extract of *Potentilla pedata* was the only Rosoideae subfamily plant determined to contain a higher concentration of chlorogenic acid.

In respect the presence of other phenolic compounds (Figure 2), the PyCo extract was found to contain a high concentration of rutin, while PoHi was determined to be the only extract containing the molecule of gallocatechin.

### 2.3. Antioxidant Capacity Evaluation

**Figure 3:**
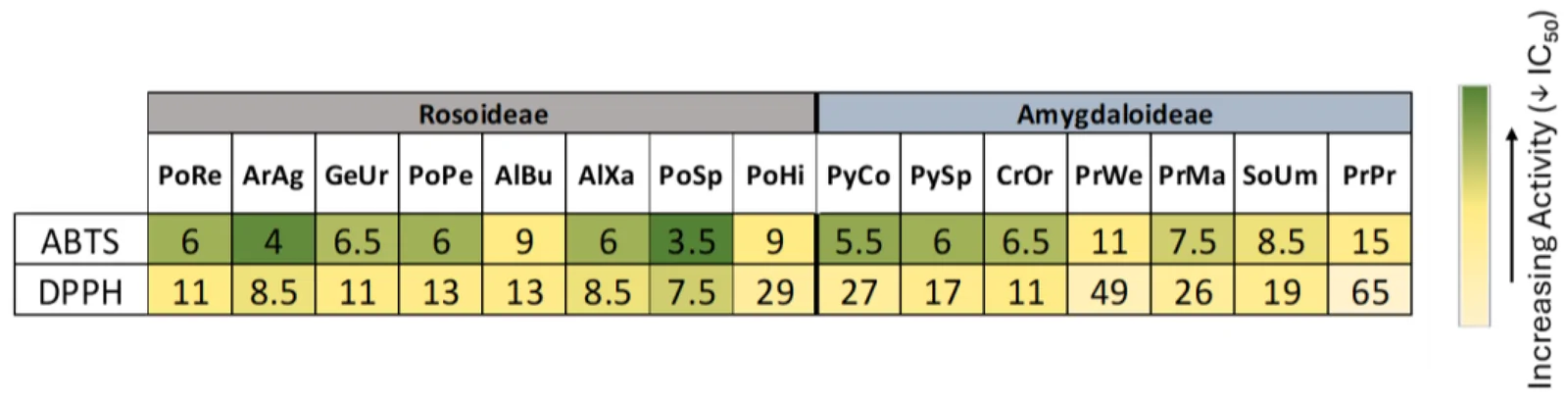
Heatmap representation of the antioxidant activity of the methanolic extracts of studied plants. Antioxidant activity was evaluated using ABTS and DPPH^•^ assays. Data are expressed as IC_50_ values (μg/mL). Green indicates increased activity (lower IC_50_), whereas yellow/beige indicates reduced activity (higher IC_50_).

The antioxidant capacities of the investigated extracts were evaluated using ABTS and DPPH^•^ assays. The respective results are included in Figure 3; these indicate that the investigated Rosoideae subfamily plants demonstrated overall a higher antioxidant activity compared to those belonging to Amygdaloideae subfamily. Specifically, the PoSp extract was found to possess in both assays the highest antioxidant activity, displaying IC_50_ values of 3.5 and 7.5 μg/mL. The ArAg extract was determined to be the next-most active, exhibiting IC_50_ values of 4.5 and 8.5 μg/mL.

### 2.4. Evaluation of Antiviral Activity

**Figure 4:**
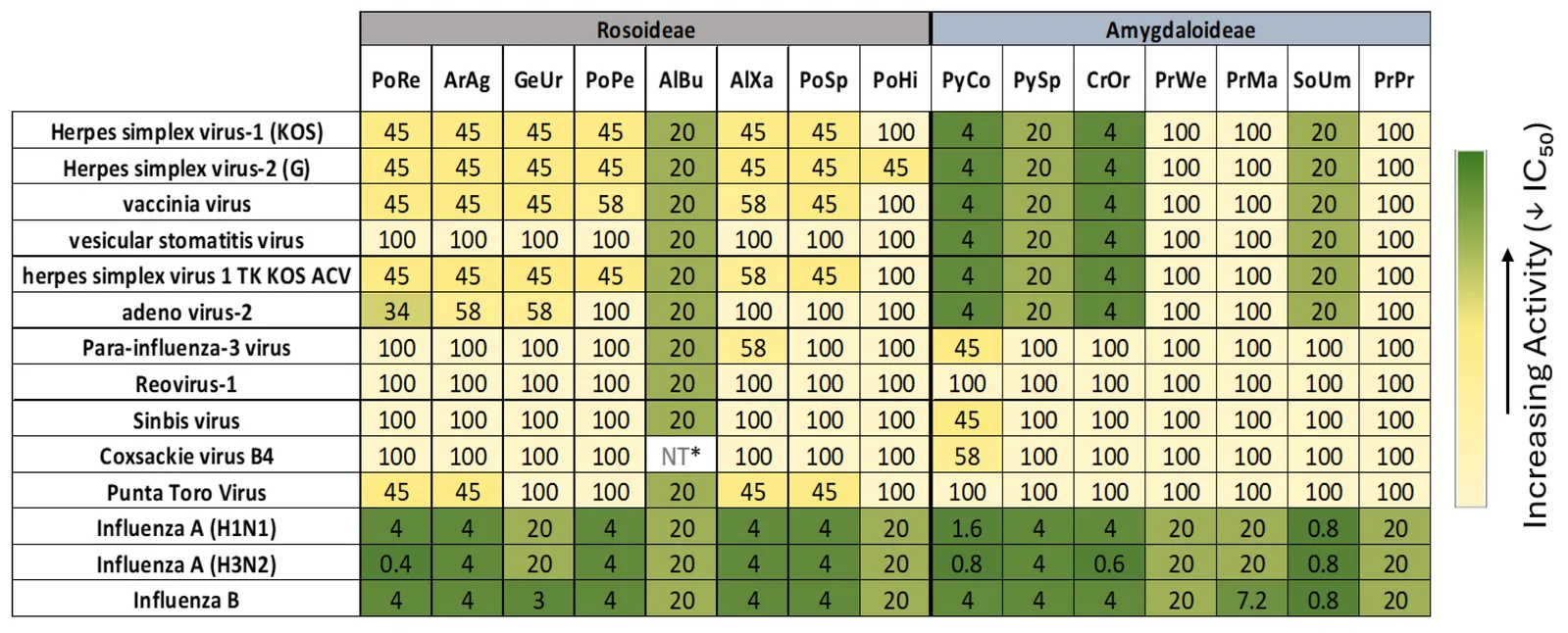
Representation of a heatmap showing antiviral activity of the methanolic extracts of the plants studied. Data are expressed as IC_50_ values (μg/mL). Green indicates increased activity (lower IC_50_), whereas yellow/beige indicates reduced activity (higher IC_50_). Values reported as 100 μg/mL correspond to extracts classified as inactive under the specific experimental conditions. * NT (Not Tested) indicates not tested due to insufficient extract quantity.

The antiviral potency of the investigated plant species was evaluated against the following fourteen virus strains: herpes simplex virus-1 (KOS), herpes simplex virus-2 (G), vaccinia virus, vesicular stomatitis virus, herpes simplex virus 1 TK KOS, adenovirus-2, parainfluenza-3 virus, reovirus-1, Sindbis virus, coxsackievirus B4, and Punta Toro virus, as well as the influenza viruses type A (H1N1 and H3N2) and type B. The respective results, presented as heatmap and IC_50_ values, are included in Figure 4.

Interestingly, both investigated plant subfamilies extracts were determined to exhibit notable activity against all influenza viruses tested. The most active was determined to be the SoUm extract, which displayed an IC_50_ value 0.8 μg/mL against all tested strains, while PoRe, CrOr and PyCo exhibited selective activity against the H3N2 strain, recording IC_50_ values 0.4, 0.6 and 0.8 μg/mL, respectively. With respect to the remaining eleven viruses, the investigated Rosoideae subfamily plant extracts exhibited moderate to low activity, with the AlBu extract displaying relatively low IC_50_ values against all tested viruses. On the contrary, the Amygdaloideae extracts PyCo, CrOr, PySp and SoUm demonstrated antiviral activity against six tested viruses, with PyCo and CrOr displaying relatively low IC_50_ values and therefore requiring further investigation.

## 3. Discussion

The results revealed variation in both total phenolic and flavonoid contents, as well as in the presence of individual phenolic compounds, among the studied extracts. In particular, the determined TPC values ranged from 169.3 to 541.3 mg GAE/g DW, with the highest value recorded for PrWe, followed by SoUm and CrOr, whereas PoHi exhibited the lowest TPC value. Regarding calculated TFC values, these displayed a broader variation, ranging from 8.9 mg QE/g DW for PoHi to 116.1 mg QE/g DW for SoUm. Interestingly, the TFC value fluctuation was not consistently parallel to the corresponding values. This observation can be rationalized by considering that differences in total phenolic content are not associated solely with flavonoid accumulation but also arise from the cumulative contribution of phenolic acids, flavonoids, and other phenolic constituents. Similarly, the determination of individual phenolic content profiles indicated a considerable chemical heterogeneity.

Specifically, the quantitation of individual phenolic acids revealed that the Amygdaloideae subfamily plant extracts are characterized by a high content of chlorogenic acid, whereas extracts of Rosoideae subfamily plants mainly contain large amounts of ellagic acid. These findings are in agreement with reports in the literature concerning the presence of individual phenolic compounds in Rosaceae family plants. Thus, the phenolic profile of the aerial parts of *Pyracantha coccinea* (PyCo) reported by Sharifi-Rad et al. (2021) reveals the presence of 28 phenolic acids, flavonoids, and lignans, highlighting chlorogenic acid as the most abundant phenolic acid and kaempferol as the predominant flavonoid [11]. In the present study, although the chlorogenic acid was determined to be the most abundant phenolic acid, the molecule of kaempferol was not detected. On the other hand, the *Pyrus spinosa* (PySp) extract was determined herein to exert an exceptionally high level of chlorogenic acid (35,173.6 mg/100 g DW), which was highlighted as the predominant molecule. The study by Kotsou et al. (2024) of fruit extracts of *Pyrus spinosa* (PySp), also known as wild pear, identified as major components the molecules of chlorogenic acid, catechin and rutin [12]. Another extract possessing an elevated content of chlorogenic acid (3989.1 mg/100 g DW) is *Prunus webbii* (PrWe), a plant previously investigated only for its amygdalin content [13,14]; however, there are no comprehensive studies on its phenolic profile.

Similarly, ellagic acid was present in large amounts in the *Potentilla speciosa* (PoSp) and *Alchemilla xanthochlora* (AlXa) extracts, at concentrations of 4127.0 and 2226.9 mg/100 g DW, respectively. It must be noted that the Uysal et al. (2017) investigation of PoSp phytochemical content also revealed the presence of gallic acid, catechin, rutin, sinapic acid, and quercetin [15]. The extract of *Prunus prostrata* (PrPr) investigated herein was characterized by a relatively high concentration of quercetin, caffeic acid, *p*-coumaric acid and ferulic acid, whereas the *Aremonia agrimonoides* (ArAg) extract showed a comparatively high catechin content (386.1 mg/100 g DW), along with a large amount of ellagic acid (714.3 mg/100 g DW). A previous investigation of PrPr aerial parts by Bilia et al. (1996) revealed the presence of kaempferol, catechin and catechin derivatives [16]. Finally, the extract of *Potentilla haynaldiana* (PoHi) was distinctive among all investigated extracts because of its high gallocatechin (243.1 mg/100 g DW) and gallic acid (61.9 mg/100 g DW) content, although this extract exhibited the lowest overall TFC and TPC values. It should be noted that the PoHi plant has not been investigated until now, and this study constitutes the first report for this species. Differences between the results obtained herein and those reported in the literature may partially be attributed to differences in the employed extraction solvents. The present study refers to the investigation of methanolic extracts, whereas previous studies have predominantly evaluated the aqueous or ethanolic extracts. Because the extraction solvent may markedly influence the qualitative and quantitative composition of plant extracts, particularly their phenolic profiles, differences in the observed biological activities may be expected. 

**Figure 5:**
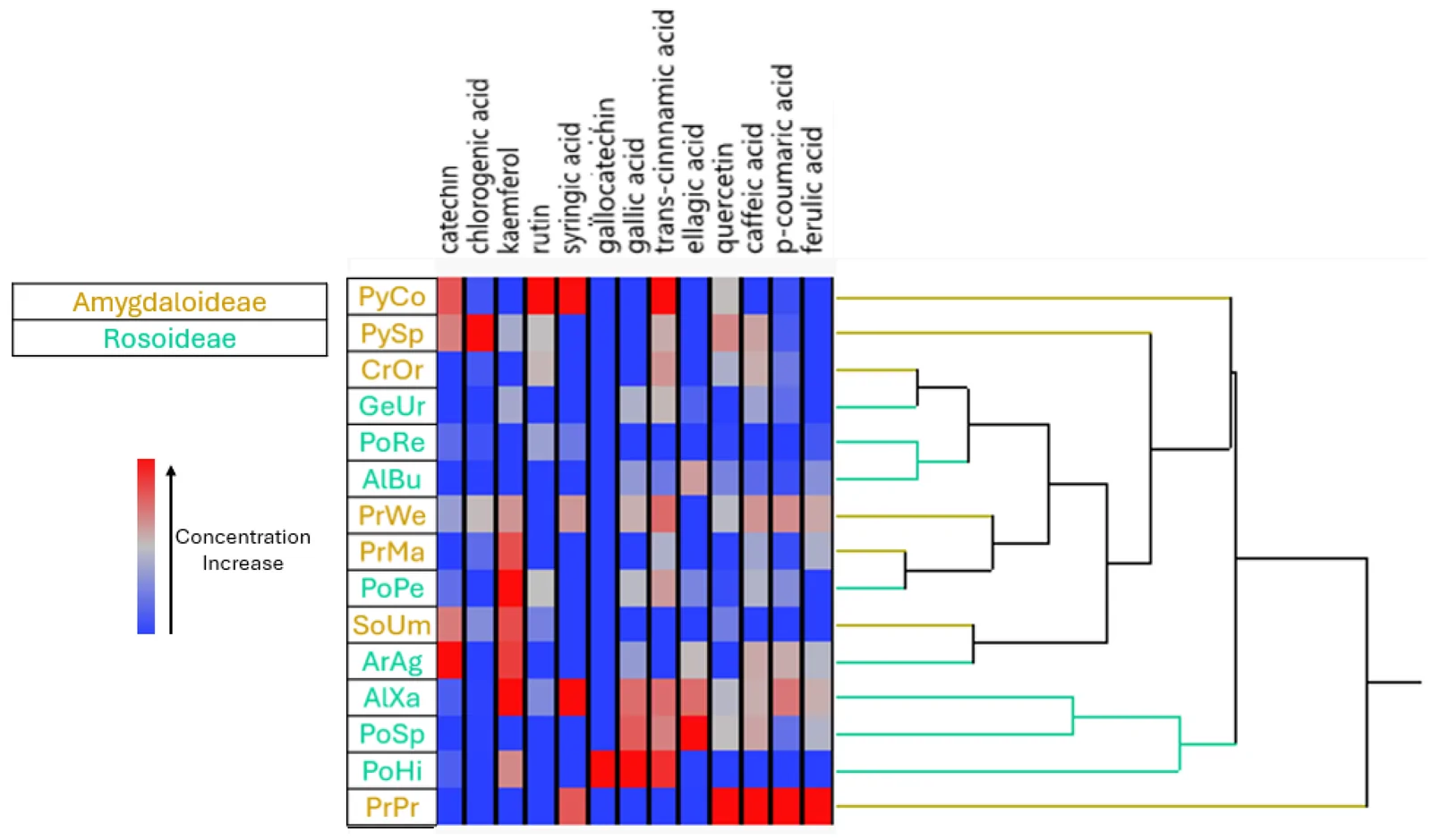
Hierarchical clustering of investigated Amygdaloideae and Rosoideae plant extracts based on their metabolite concentration profiles, with color intensities indicating relative concentration levels.

A hierarchical clustering of samples based on their metabolite concentration profiles is shown in Figure 5. Several samples exhibited relatively high concentrations of specific phenolic constituents, particularly gallic acid, caffeic acid, *p*-coumaric acid and ferulic acid, whereas other compounds, such as catechin, chlorogenic acid and rutin, showed a more heterogeneous distribution across the samples. This variation indicates that the analyzed taxa may differ not only in their total abundance of phenolic compounds but also in the relative contribution of individual metabolites to their chemical profiles. The hierarchical clustering further demonstrated that samples with similar phenolic profiles tended to cluster together, supporting the potential usefulness of these compounds as chemotaxonomic markers. In this respect, some closely grouped extracts showed comparable patterns for several metabolites, whereas extracts positioned on more distant branches of the dendrogram displayed more divergent chemical compositions. This clustering pattern suggests that phenolic profiles may reflect underlying biological or taxonomic relationships, although additional parameters, such as environmental conditions and plant developmental stage may also contribute to the observed differences. Interestingly, the separation between samples belonging to the Amygdaloideae and Rosideae groups was not absolute, indicating that chemical similarity does not necessarily follow higher-level taxonomic classification. The presence of overlapping metabolite patterns between groups may reflect shared biosynthetic pathways or common ecological adaptations. Conversely, the distinct profiles observed in some samples highlight the potential for species-specific patterns of phenolic accumulation.

Overall, these findings indicate that the studied taxa differ not only in the abundance of contained phenolic compounds but also in the composition and relative contribution of individual metabolites. Such differences may reflect species-specific metabolic characteristics and differences in phenolic biosynthesis. However, because the present dataset does not include biological variability parameters or statistical comparisons among biological replicates, these differences should be considered as descriptive, rather than as solid evidence of a statistically significant variation. In particular, the use of a single biological replicate per sample limits the extent to which biological variability and statistically significant differences can be assessed. The outcome of the antioxidant capacity evaluation of investigated extracts is included in Figure 3. This reveals differences in determined antioxidant activities between the investigated Rosoideae and Amygdaloideae taxa. Although the magnitude of the determined activities differs between the ABTS and DPPH^•^ assays, both produce broadly comparable patterns, with several Rosoideae extracts showing relatively low IC_50_ values. The IC_50_ values recorded in the ABTS assay for these taxa ranged between 3.5 and 15 μg/mL, whereas the respective DPPH^•^ values fluctuated in a wider range (7.5–65). Specifically, within the Rosoideae subfamily lower ABTS values were observed for PoSp (3.5), ArAg (4.0), PoRe (6.0), PoPe (6.0) and AlXa (6.0) extracts, whereas AlBu and PoHi extracts displayed somewhat higher values (9.0). A similar trend was evidenced in the DPPH^•^ assay results for these taxa, except the result for the PoHi extract which was particularly distinctive, displaying an IC_50_ value of 29.0 μg/mL, while for the remaining Rosoideae plants this value fluctuated between 7.5–13.0 μg/mL. Nevertheless, in both assays PoSp and ArAg extracts were found to display the lowest IC_50_ values, indicating a relatively higher radical-scavenging activity under the specific conditions used for these assays. With respect to the evaluation of the antioxidant capacities of Amygdaloideae subfamily extracts, the results obtained from the two assays revealed a considerable variation among investigated taxa. Such discrepancies can be rationalized by considering that the ABTS and DPPH^•^ assays differ in their reaction mechanisms, radical characteristics, and sensitivity to different classes of antioxidant compounds. In addition, the individual phenolic compounds exhibit different reactivities against the two radical systems, depending on their chemical structure, substitution pattern, and ability to release hydrogen atoms or electrons. Consequently, phenolic constituents that contribute strongly to radical scavenging in one assay may show comparatively lower activity in the other, potentially accounting for the differences observed between the ABTS and DPPH^•^ results.

The bioactivities of the plant taxa investigated herein are included in a plethora of reports in the literature. Specifically, the ethanolic and aqueous extract of *Pyracantha coccinea* (PyCo) and *Prunus mahaleb* (PrMa) were found to possess a significant antioxidant activity in [17]. Additional studies reporting the in vivo anti-inflammatory and antinociceptive properties of PyCo leaf extracts verified their capability to reduce carrageenan-induced paw edema and the formalin-induced pain [18]. Another study concerning the cytotoxic and antimicrobial activities of *Pyrus spinosa* (PySp) fruit, leaf and bark extracts revealed their potent antibacterial and antifungal activities, with bark extracts showing cytotoxicity toward the Fem-x melanoma cell line [19]. The leaves and berries of *Crataegus orientalis* (CrOr) were investigated for their antioxidant potency, and it was found that leaf extracts displayed a potent DPPH-scavenging and reducing activity, with both leaf and berry extracts being found to inhibit various inflammatory pathways [20]. The fruits, leaves, twigs, seeds, and kernels of *Prunus mahaleb* (PrMa) were also found to demonstrate potent antioxidant and tyrosinase-inhibitory activities, with twig, seed and kernel extracts exhibiting particularly strong effects, including antimelanoma activity [21,22]. *Sorbus umbellata* (SoUm) extracts were determined to possess anticholinesterase activity, while the molecules of chlorogenic acid and rutin were identified as the active components of the extracts [23]. In addition, SoUm leaf extracts were shown a potent antiproliferative activity against A549 lung cancer cells and antibacterial activity against *Staphylococcus aureus* [24]. Finally, ethyl acetate extracts of *Geum urbanum* (GeUr) exhibited strong antibacterial activity against Gram-positive bacteria, including *Staphylococcus* spp. and *Bacillus cereus*, along with potent radical-scavenging activity [25]. Contrarily, reports on the antiviral activity of these taxa are scarce, because only GeUr extracts have been tested against HSV-1, adenovirus-5, poliovirus, Coxsackie B1/B3 and RSV, demonstrating activity only against the HSV-1 and HAdV-5 strains [26]. Moreover, a 2021 study concerning the water extract of *Prunus mahaleb* fruits reported its capability to down-regulate the expression of ACE2 and TMPRSS2 in H1299 cells. This finding was interpreted as an indication of its potential to inhibit entry of SARS-CoV-2 into host cells [27]. Herein, the antiviral activities of the investigated methanolic extracts are presented as a heatmap in Figure 4, in which it can be seen that they all displayed pronounced activity against all tested influenza viruses. Specifically, the SoUm and PyCo extracts showed the lowest observed IC_50_ values against the H1N1 strain, displaying respectively IC_50_ values 0.8 and 1.6 μg/mL. On the contrary, several extracts exhibited a potent activity against the H3N2 strain, with PoRe, CrOr, PyCo and SoUm extracts with IC_50_ values of 0.4, 0.6, 0.8 and 0.8 μg/mL, respectively. Finally, the SoUm extract showed the lowest observed IC_50_ value against the influenza B strain, exhibiting an IC_50_ value of 0.8 μg/mL.

With respect to the remaining eleven virus strains tested, the methanolic extracts of PyCo and CrOr showed measurable activity against herpes simplex virus type 1 (KOS), herpes simplex virus type 2 (G), vaccinia virus, vesicular stomatitis virus, HSV-1 TK KOS ACV, and adenovirus type 2. Additionally, PyCo extract exhibited moderate activity against parainfluenza virus type 3, Sindbis virus, and coxsackievirus B4. These results indicate that some extracts displayed activity across multiple viral models. However, direct comparisons with purified antiviral agents are not appropriate without equivalent cytotoxicity, selectivity, and assay-validation data.

The anti-influenza activities observed herein are of particular interest because they refer to important human pathogens. In addition, the recently observed antiviral resistance and the continual evolution of circulating strains underscore the need for the development of novel antiviral agents [28]. Thus, the recorded antiviral provide preliminary evidence suggesting that the investigated plant extracts contain constituents associated with antiviral activity under the tested conditions. However, these findings should be interpreted as preliminary evidence of antiviral activity rather than proof of therapeutic efficacy. Further studies using replicated experiments, dose-response analyses, cytotoxicity assays, and isolation and characterization of the active constituents are necessary to determine the reproducibility, selectivity, and mechanisms underlying the observed antiviral effects.

## 4. Materials and Methods

### 4.1. Solvents, Chemicals and Standards

The extractions were performed using as solvents methanol, ethanol, n-hexane and dimethylsulfoxide (DMSO), all purchased as analytical grade chemicals from Fisher Chemicals (Hampton, NH, USA). The chemicals sodium carbonate (anhydrous), sodium hydroxide, sulfuric acid (98%), potassium hydroxide (all >98%) and hydrochloric acid were supplied by Chem-Lab (Zedelgem, Belgium), while ferric chloride hexahydrate, sodium nitrite, and iron sulfate heptahydrate were obtained from Alfa Aesar (Heysham, UK). For chromatographic separations, LC-MS grade water and acetonitrile obtained from Merck (Darmstadt, Germany) were utilized. The Folin–Ciocalteu reagent, 2,2-diphenyl-1-picrylhydrazyl (DPPH), 2,2′-azino-bis(3-ethylbenzthiazoline-6-sulfonic acid) (ABTS), hydrogen peroxide (H_2_O_2_) and horseradish peroxidise (HRP) were purchased from Sigma-Aldrich (Burlington, MA, USA), while the acetic acid and aluminum chloride hexahydrate were provided by Sigma-Aldrich (Burlington, MA, USA). All standards used for the determination of the contained phytochemicals were provided by Sigma-Aldrich (Burlington, MA, USA), except catechin and gallic acid, which were purchased from ExtraSynthese (Genay, France). The PTFE filters (0.45 μm) used for extract’s filtration were provided by Macherey-Nagel (Duren, Germany).

### 4.2. Plant Material Sample Extraction

The aerial parts of the investigated plants were collected during the summer period of 2024 from the respective wild plants’ colonies growing on various mountains of Greece. The detailed collection data are included in Table 1. A voucher specimen for each sampling material has been deposited in the herbarium of the Agricultural University of Athens.

After sampling, an amount of 500 g from each sample was placed into a covered, well-ventilated room and allowed to dry for three months in the absence of light. The dried samples were cut into small pieces and grounded with the aid of a POLYMIX, PX-MFC 90 D laboratory scale mill (KINEMATICA, Lucern, Switzerland). The obtained powdered material was transferred into a glass flask containing 1L of hexane and stirred in the absence of light for 48 h to remove the contained lipids and chlorophyll. The solvent was removed by filtration, the resulting solid was poured into 1 L of analytical-grade methanol, and the resulting mixture was stirred in the absence of light for 48 h. After filtration, the remaining solid was re-extracted two additional times following the same procedure. The methanolic extracts were combined and the solvent was removed under reduced pressure, temperature being maintained below 35 °C with the aid of a Büchi Rotavapor R-210 apparatus equipped with a Büchi vacuum pump V-700 and vacuum controller V-850 (all obtained from Büchi Labortechnik AG, Flawil, Switzerland), in combination with a Julabo F12 cooling unit (Julabo GmbH, Seelbach, Germany). The remaining slurry was dried to provide an amorphous powder which was placed in glass vials and kept at −19 °C prior to determination and analysis of bioactivities.

### 4.3. Determination of Total Phenolic Content (TPC) and Total Flavonoid Content (TFC)

The extracts’ TPCs were determined using the Folin–Ciocalteu colorimetric assay following a method previously developed by Singleton et al. [29]. Briefly, 100 μL of a methanolic solution from each investigated sample were transferred into a 10 mL glass flask and 6 mL of deionized water was added. Then, 1 mL of Folin–Ciocalteu reagent was added, the flask was sealed, and the mixture was maintained for 3 min at room temperature. To this mixture was added, consecutively, 1.5 mL of 20% *w*/*v* carbonate solution sodium and 10 mL of deionized water. The resulting solution was incubated in the absence of light at room temperature for 2 h. After incubation, absorbance was determined at 765 nm against a blank containing a mixture of Folin–Ciocalteu reagent and distilled water with the aid of a spectrophotometer (Hitachi U-1900, Tokyo, Japan). All measurements were performed in triplicate, and all respective results were obtained as a mean value expressed as milligrams of gallic acid equivalents (GAE) per gram of dried sample (DW).

The TFC values of the investigated samples were assessed by applying a modified version of the colorimetric assay developed by Jia et al. [30]. Briefly, 1 mL of methanolic solution of each investigated sample was placed in a 10 mL flask to which 4 mL deionized water and 0.3 mL of 5% sodium nitrite solution was then added. After maintaining the mixture at room temperature for 5 min, 0.3 mL of 10% ethanolic solution AlCl3·6H2O was added and the mixture was kept for 1 min. Then, 2 mL of a 1M potassium hydroxide solution was added, and the final volume was adjusted to 10 mL with the addition of deionized water. Subsequently, absorbance was measured at 510 nm, and the respective values were calculated against a respective blank solution. Each sample was processed in triplicate, and all outcomes were obtained as a mean value expressed of quercetin equivalents (QE) per gram of dried sample (DW).

### 4.4. Determination and Quantification of Individual Phenolic Compounds by HPLC and UPLC–MS/MS Analysis

Profiling of the presence of individual phenolic compounds in investigated samples was performed by HPLC analysis, applying a method described previously by Kerasioti et al. [31], using a Hewlett Packard HP1100 instrument (Waldbronn, Germany) equipped with a quaternary pump, a degasser and an Agilent 1100 diode-array detector (DAD) (Agilent Technologies, Santa Clara, CA, USA). Absorbance was monitored over the full wavelength range during the entire run, controlled with the ChemStation software package, version 5.01 (Hewlett Packard, Palo Alto, CA, USA). Chromatographic separation was performed using a Zorbax Eclipse Plus C18 column (5 μm particle size, 150 × 4.6 mm i.d.; Agilent Technologies, Santa Clara, CA, USA) protected by a guard column of the same stationary phase (8 × 4 mm). Samples were introduced into the system through a Rheodyne injection valve (model 7725I) fitted with a 20 μL fixed-volume loop. For each plant sample, a single extract preparation was evaluated, and one injection was performed for each extract. Therefore, the HPLC-DAD data represent single analytical measurements from a single extract preparation for each sample. The utilized HPLC-grade water was obtained from a Milli-Q purification system (Merck Millipore, Burlington, MA, USA). Except for acetonitrile, all HPLC-grade solvents were filtered through 0.45 μm cellulose acetate membrane filters before use. The mobile phase consisted of solvent A (0.3% acetic acid in water) and solvent B (acetonitrile). Separation was performed at a constant flow rate of 1 mL/min using a gradient elution program. Solvent B was initially set at 25% and maintained for 5 min, followed by 30% for 5 min, 40% for 5 min, 50% for 10 min, and 70% for 10 min. The 70% proportion was then maintained for an additional 5 min. The mobile phase was subsequently returned to the initial composition over 2 min and maintained for 15 min to equilibrate the column before the next injection. The phenolic compounds detected by HPLC-DAD were identified and quantified using authentic reference standards and external calibration curves. Identification by HPLC-DAD was based on comparison of the retention times and UV absorption characteristics of the detected peaks with those of the corresponding reference standards. Quantification was performed at the characteristic wavelengths of each compound: 280 nm for gallic acid, gallocatechin, catechin, syringic acid, ellagic acid, and trans-cinnamic acid; 320 nm for chlorogenic acid, caffeic acid, *p*-coumaric acid, and ferulic acid; and 360 nm for quercetin, kaempferol, and rutin. The identity of the detected compounds was additionally confirmed by HPLC-MS/MS analysis using Selected Ion Monitoring (SIM) and Multiple Reaction Monitoring (MRM) techniques. Thus, HPLC-MS/MS served as an additional confirmatory technique, whereas the quantitative determination of the phenolic compounds was performed exclusively by HPLC-DAD, using authentic reference standards.

### 4.5. Evaluation of Antioxidant Potencies

The antioxidant potencies of samples studied were assessed with two assays, namely, the 2,2-DiPhenyl-1-PicrylHydrazyl (DPPH^•^) and the 2,2′-Azino-Bis(3-ethylbenzoThiazoline-6-Sulfonic acid) radical scavenging (ABTS^•+^) [32,33]. The respective samples were obtained by dissolving 200 mg of each plant extract into 1 mL of methanol, mixture stirring for 20 min at ambient temperature, centrifuging, and obtaining the supernatant which was used for the antioxidant potency assessment. Each assay was repeated in triplicate, and results obtained were presented as the calculated mean value ± standard deviation.

Specifically, the DPPH^•^ assay was performed by dissolving into 1.0 mL of freshly prepared 100 μM methanolic solution of DPPH^•^ various amounts of the investigated plant extracts dissolved in methanol. The resulting mixtures were incubated for 20 min at room temperature and the absence of light. Subsequently, absorbance was recorded at 517 nm utilizing a Hitachi U-1900 ratio-beam spectrophotometer (Hitachi, Tokyo, Japan) against a blank consisting of methanol and a control DPPH^•^ solution.

The ABTS^•+^ radical scavenging activity was evaluated according to a modified version of a previously developed procedure [33]. Specifically, the ABTS^•+^ radical was generated by dissolving into 1 mL of distilled water 2 mM of ABTS with 30 μM H2O2 and 6 μM horseradish peroxidase (HRP) enzyme. The resulting mixture was incubated at ambient temperature for 45 min in the absence of light to allow the formation of ABTS^•+^ radical. Subsequently, 10 μL of methanolic solutions containing various amounts of investigated plant extracts were added, and the absorbance of the resulting solution was recorded at 730 nm utilizing a Hitachi U-1900 ratio beam spectrophotometer (Tokyo, Japan) against a blank consisted of distilled water containing ABTS and H_2_O_2_, and an ABTS•+ radical solution in 10 μL H_2_O utilized as control.

For both assays, the percentage of the radical scavenging capacity (RSC) of investigated plant extracts was assessed with the aid of the following equation:

Radical scavenging capacity (%) = [(A_control_ − A_sample_)/A_control_] × 100

where A_control_ and A_sample_ are the absorbance values of the control and the tested sample, respectively.

The IC_50_ (Inhibitory Concentration), defined as the concentration of the tested extract required to achieve 50% inhibition (radical scavenging) of DPPH^•^ or ABTS^•+^ radicals, was determined by linear regression analysis of the radical scavenging capacity (RSC, %) as a function of extract concentration. The corresponding regression equations were used to calculate the concentration associated with 50% radical inhibition. Statistical analyses and regression calculations were performed using SPSS software, version 13.0. The IC_50_ values, expressed in μg/mL, represent the mean of three independent experimental replicates. Lower IC_50_ values indicate stronger radical scavenging and, consequently, higher antioxidant activity of the extracts.

### 4.6. Evaluation of Antiviral Properties

The antiviral properties of the investigated plant extracts were assessed against the following panel of viral pathogens: herpes simplex virus type 1 (HSV-1) strain KOS, herpes simplex virus type 2 strain G, vaccinia virus Lederle strain, vesicular stomatitis virus (VSV), herpes simplex virus type 1 (TK KOS ACV), adenovirus-2, parainfluenza virus 3, reovirus-1, Sindbis virus, coxsackie virus B4, Punta Toro virus, influenza virus A (subtypes H1N1, H3N2) and influenza virus B. Antiviral activities were evaluated according to previously reported procedures [34], based on inhibition of virus-induced cytopathicity and/or viral plaque formation in susceptible cell cultures. Briefly, cells were infected with the appropriate virus inoculum (typically 100 CCID_50_, or 100 PFU/well for plaque-based assays) and exposed to five-fold serial dilutions of the test extracts. Viral cytopathicity or plaque formation was assessed after incubation when the effect was maximal in untreated infected controls. For HCMV, HEL fibroblasts were infected with 100 PFU/well and incubated for 7 days at 37 °C, whereas HIV assays were performed in CEM cells using 100 CCID_50_/mL and 4–5 days of incubation. Antiviral activity is expressed as IC_50_, defined as the concentration required to reduce virus-induced cytopathogenicity or viral plaque formation by 50% relative to the corresponding untreated viral control. Uninfected cells were included to assess cytotoxicity and distinguish specific antiviral effects from nonspecific effects on cell viability.

### 4.7. Data Analysis

The statistical analyses were performed using Microsoft Excel 365 and JMP^®^ Student Edition software (Edition 18). For quantification of the presence of individual phenolic compounds, the respective calibration curves were constructed using linear regression analysis in Microsoft Excel. Hierarchical cluster analysis was performed as an exploratory analysis in JMP Student Edition using Ward’s method with squared Euclidean distance. Prior to clustering, metabolite concentrations were standardized by column using z-score standardization. Robust standardization was not applied. Compounds that were not detected were assigned a value of zero, and no missing-value imputation was performed. Because only one biological sample was available for each taxon, the hierarchical clustering analysis was considered exploratory and was used to visualize patterns of similarity in metabolite profiles rather than to assess statistically significant differences or biological variability among taxa. Therefore, the observed clustering patterns should be interpreted as descriptive and indicative of potential associations which require confirmation in future studies using independent biological replicates.

### 4.8. Use of Generative Artificial Intelligence

Generative artificial intelligence (ChatGPT, OpenAI) was used to generate selected illustrative visual elements for the graphical abstract. The final composition, layout, and integration of these elements were carried out by the authors using Microsoft PowerPoint 365. The AI-generated elements were reviewed by the authors to ensure that the final graphical abstract accurately represents the study.

## 5. Conclusions

The exploratory study presented herein revealed the rich phytochemical profile of investigated fifteen Rosaceae species, highlighting their potential to act as rich sources of bioactive natural products. The methanolic extracts of the investigated taxa showed variation in their total phenolic and flavonoid contents and in their individual phenolic profiles, with chlorogenic acid and ellagic acid representing the most abundant molecules. Their chemical diversity was accompanied by variation in antioxidant activity, with *Potentilla speciosa* and *Aremonia agrimonoides* displaying the lowest IC_50_ values in the two radical-scavenging assays. Antiviral screening provided preliminary evidence of their activity, particularly against the influenza A and B viruses. The extracts of *Pyracantha coccinea* and *Crataegus orientalis* were active against multiple viruses, while relatively low IC_50_ values against influenza A viruses were recorded for the extracts of *Potentilla recta*, *Crataegus orientalis*, *Pyracantha coccinea* and *Sorbus umbellata*. These findings are of particular interest because several of the investigated taxa, especially within the Rosoideae subfamily, are relatively poorly characterized from phytochemical and antiviral perspectives. Overall, these preliminary results provide a basis for a more precise evaluation of these underexplored Rosaceae species in respect to their phytochemical content and pharmacological activities, with an emphasis on antiviral and antioxidant properties.

## Figures and Tables

**Table 1:** Rosaceae plant material sampling data.

Code	Taxa	Subfamily	Location	Altitude	Coordinates
PyCo	*Pyracantha coccinea*	Amygdaloideae	Mt. Parnitha	1048	38°08.371′ N 023°44.510′ E
PySp	*Pyrus spinosa*	Amygdaloideae	Mt. Parnitha	580	38°19.054′ N 023°22.955′ E
CrOr	*Crataegus orientalis*	Amygdaloideae	Mt Giona	1386	38°58.305′ N 022°43.883′ E
PrWe	*Prunus webbii*	Amygdaloideae	Mt Parnitha	1006	38°09.332′ N 023°43.193′ E
PrMa	*Prunus mahaleb*	Amygdaloideae	Mt Chelmos	1675	37°39.054′ N 022°22.955′ E
SoUm	*Sorbus umbellate*	Amygdaloideae	Mt Chemlos	1583	37°41.233′ N 022°13.634′ E
PrPr	*Prunus prostrata*	Amygdaloideae	Mt Tymfi	1483	39°49.332′ N 021°43.193′ E
PoRe	*Potentilla recta*	Rosoideae	Mt Chelmos	1621	37°49.920′ N 022°16.321′ E
ArAg	*Aremonia agrimonoides*	Rosoideae	Mt Oiti	1777	38°49.673′ N 022°16.368′ E
GeUr	*Geum urbanum*	Rosoideae	Mt Oiti	1245	38°20.865′ N 022°13.315′ E
PoPe	*Potentilla pedate*	Rosoideae	Mt Oiti	1614	38°25.753′ N 022°11.515′ E
AlBu	*Alchemilla bulgarica*	Rosoideae	Mt Oiti	1896	38°49.246′ N 022°16.433′ E
AlXa	*Alchemilla xanthochlora*	Rosoideae	Mt Oiti	1571	38°46.153′ N 022°15.331′ E
PoSp	*Potentilla speciosa*	Rosoideae	Mt Oiti	1765	38°50.106′ N 022°16.221′ E
PoHi	*Potentilla haynaldiana*	Rosoideae	Mt Chelmos	1602	37°58.332′ N 022°23.131′ E

## Data Availability

The original contributions presented in this study are included in the article. Further inquiries can be directed to the corresponding author.

## Supplementary Materials

The following supporting information can be downloaded at https://www.mdpi.com/article/10.3390/molecules31203651/s1. Table S1-A. Total phenolic content (TPC) expressed as mg GAE/dried weight extract and total flavonoid content (TFC) expressed as mg QE/dried weight extract of studied Amygdaloideae plant species. Table S1-B. Total phenolic content (TPC) expressed as mg GAE/dried weight extract and total flavonoid content (TFC) expressed as mg QE/dried weight extract of studied Rosoideae plant species. Table S2. Quantitation of presence of phenolic compounds in the Amygdaloideae and Rosoideae plant species investigated expressed as mg/100 g of their dried methanolic extracts. Table S3. Activity of reference antiviral agents against eleven viruses, expressed as IC_50_ values (μg/mL). Table S4. Activity of reference antiviral agents against infuenza viruses, expressed as IC_50_ values (μg/mL). Table S5. Classification of antiviral activity according to IC_50_ values. Table S6. Search terms used in the Scopus database and the corresponding number of retrieved publications. Table S7. Calibration parameters and retention times of the individual phenolic compounds determined by HPLC-DAD.
