# Supplementary material for: Preliminary Assessments of Phenolic Profiles, Antioxidant Capacities, and Antiviral Activities of Fifteen Rosaceae Species
Source: Molecules. 2026 Oct 13;31(20):3651. doi: 10.3390/molecules31203651 (PMC13707888; doi:10.3390/molecules31203651)
Supplement: Supplementary file 1 [file molecules-31-03651-s001.zip › molecules-4614829-supplementary.pdf]

Supplementary Table S1-A. Total phenolic content (TPC) expressed as mg GAE/dried weight extract and total flavonoid content (TFC) expressed as mg QE/dried weight extract of studied Amygdaloideae plant species.

	Pyracantha coccinea	*Pyrus spinosa*	*Crataegus orientalis*	*Prunus webii*	*Prunus mahaleb*	*Sorbus umbellata*	*Prunus prostrata*
TPC	369.00±0.00	438.67±28.6	513.33±22.23	541.35±57.40	310.67±12.76	520.43±6.13	205.00±17.28
TFC	92.56±.7.29	58.78±2.08	96.89±17.78	62.67±7.45	95.02±8.62	116.14±5.12	26.11±0.52

Supplementary Table S1-B. Total phenolic content (TPC) expressed as mg GAE/dried weight extract and total flavonoid content (TFC) expressed as mg QE/dried weight extract of studied Rosoideae plant species.

	Potentilla recta	*Aremonia agrimonoides*	*Geum urbanum*	*Potentilla pedata*	*Alchemilla bulgarica*	*Alchemilla xanthochlora*	*Potentilla speciosa*	*Potentilla haynaldiana*
TPC	307.67±7.04	367.03±156.03	323.08±131.05	406.67±34.10	298.01±12.19	424.33±15.80	404.33±31.93	169.33±14.38
TFC	48.33±4.46	51.67±24.83	23.33±11.47	56.56±6.32	26.06±3.54	44.12±2.51	28.45±0.95	8.89±1.00

Supplementary Table S2. Quantitation of presence of phenolic compounds in the Amygdaloideae and Rosoideae plant species investigated expressed as mg/100 g of their dried methanolic extracts

	Catechin	Rutin	Kaemferol	Gallic acid	Gallocatechin	Chlorogenic acid	Caffeic acid	Syringic acid	*p*-Coumaric acid	Ferulic acid	Ellagic acid	Quercetin	*trans*-Cinnamic acid
Pyracantha coccinea	261,2	1634.8	ND	ND	ND	457.9	ND	18.3	0.6	ND	ND	16.0	8.7
Pyrus spinosa	188,9	186.3	2.1	ND	ND	35173.6	8.4	ND	1.1	ND	ND	38.2	4.4
Crataegus orientalis	ND	253.0	ND	ND	ND	516.3	7.4	ND	2.2	ND	ND	12.4	5.0
Prunus webbii	57.4	ND	3.4	17.8	ND	3989.1	9.7	7.1	11.6	13.7	ND	13.7	6.1
Prunus mahaleb	ND	ND	4.8	ND	ND	989.6	4.5	ND	0.5	5.9	ND	0.5	3.4
Sorbus umbellata	195.6	86.7	5.0	ND	ND	1723.8	ND	ND	ND	ND	ND	6.9	ND
Prunus prostrata	ND	ND	ND	ND	ND	Tr	21.8	12.2	28.5	53.7	ND	90.6	ND
Potentilla recta	26.5	128.1	ND	ND	ND	415.7	ND	1.7	ND	1.3	ND	0.8	ND
Aremonia agrimonoides	386.1	ND	5.1	9.4	ND	Tr	7.8	ND	7.7	6.3	714.3	ND	ND
Geum urbanum	ND	ND	2.0	11.5	ND	Tr	4,4	ND	1.8	ND	153.5	ND	4.1
Potentilla pedate	28.6	171.9	6.1	12.5	ND	Tr	5.2	ND	3.0	ND	312.5	1.4	4.9
Alchemilla bulgarica	ND	ND	ND	9.2	ND	Tr	1.9	ND	0.6	4.2	1281.5	7.6	1,7
Alchemilla xanthochlora	18.3	98.7	6.1	35.7	ND	71.4	7.4	18.5	14.5	11.6	2226.9	13.4	6.1
Potentilla speciosa	ND	ND	Tr	40.1	ND	45.6	8.3	ND	1.9	6.2	4127.0	15.3	5.6
Potentilla haynaldiana	18.9	ND	3.6	61.9	243.1	26.9	ND	ND	ND	ND	8.3	ND	7.8

ND: NotDetected; Tr: Trace

Supplementary Table S3. Activity of reference antiviral agents against eleven viruses, expressed as IC_{50} values (g/mL).

Taxa	Herpes simplex virus-1 (KOS)	Herpes simplex virus-2G	Vaccinia virus	Vesicular stomatitis virus	Herpes simplex virus-1 TK (KOS)	Adenovirus -2	Parainfluenza-3 virus	Reovirus-1	Sindbis virus	Coxsackie virus B4	Punta Toro virus
Bivudin	>250	>250	25	>250	50	-	-	-	-	-	-
Cidofovir	>250	2	22	>250	2	10	-	-	-	-	-
Acyclovir	>250	0.4	>250	>250	5.9	-	-	-	-	-	-
Ganciclovir	>100	0.03	>100	>100	0.8	-	-	-	-	-	-
DS-10.000	4	20	4	100	100	20	>100	>100	100	34	100
Ribavirin	4	20	4	100	100	20	146	112	>250	>250	112

Supplementary Table S4. Activity of reference antiviral agents against infuenza viruses, expressed as IC_{50} values (g/mL).

	Zanamivir	Ribavirin	Amantadine
Influenza A H1N1	0.1	8.5	4.4
Influenza A H3N2	1.5	6.9	0.7
Influenza B	0.2	7.7	>200

Supplementary Table S5. Classification of antiviral activity according to IC_{50} values.

IC_{50} value (μg/mL)	Antiviral activity
< 1	Very strong / highly potent activity
1–10	Strong activity / promising activity
>10–50	Moderate activity
>50–99	Weak activity
≥100	Inactive or negligible activity

Supplementary Table S6. Search terms used in the Scopus database and the corresponding number of retrieved publications.

**Search term**	**Number of retrieved publications**
Pyracantha coccinea	110
Pyrus spinosa	64
Crataegus orientalis	128
Prunus webbii	30
Prunus mahaleb	487
Sorbus umbellata	12
Prunus prostrata	26
Potentilla recta	81
Potentilla pedata	6
Potentilla speciosa	13
Potentilla haynaldiana	0
Alchemilla bulgarica	0

Alchemilla xanthochlora	30
Aremonia agrimonoides	2
Geum urbanum	162
Fragaria ananassa	10063
Rubus idaeus	2059
Rubus fruticosus	656
Rosa damascena	1297
Rosa canina	1288
Rosa gallica	125
Potentilla	2481
Alchemilla	556
Aremonia	3
Geum	921
Fragaria	14243
Rubus	9709
Rosa	23834

Supplementary Table S7. Calibration parameters and retention times of the individual phenolic compounds determined by HPLC-DAD.

Compound	RT (min)	Calibration range (ppm)	Calibration Curve	R^2
Gallic acid	3.5	3-600	y = 55.213x + 109.58	0.993
Gallocatechin	6.3	3-350	y = 4.7615x - 72.537	0.997
Chlorogenic acid	9.1	2-700	y = 39.927x - 1701.2	0.995
Caffeic acid	9.9	1-450	y = 79.647x + 602.51	0.994
Catechin	11.0	3-600	y = 2.7078x - 86.701	0.994
Syringic acid	12.3	2-400	y = 187.99x + 9065.5	0.992
Rutin	15.7	3-1200	y = 99.297x - 12532	0.998
Ellagic acid	18.1	1.5-800	y = 5.3221x - 5.122	1.000
p-Coumaric acid	18.8	2-250	y = 9.8245x - 60.254	0.998
Ferulic acid	21.3	1-200	y = 11.637x - 114.94	0.998
Quercetin	25.4	1.5-650	y = 11.328x - 41.35	0.998
Kaempferol	27.8	3-300	y = 5.5542x - 6.4054	0.998
trans-Cinnamic acid	29.2	1.5-400	y = 28.555x - 1769.3	0.992
